# Icariin-Functionalized Nanodiamonds to Enhance Osteogenic Capacity In Vitro

**DOI:** 10.3390/nano10102071

**Published:** 2020-10-20

**Authors:** Somang Choi, Sung Hyun Noh, Chae Ouk Lim, Hak-Jun Kim, Han-Saem Jo, Ji Seon Min, Kyeongsoon Park, Sung Eun Kim

**Affiliations:** 1 Department of Orthopedic Surgery and Rare Diseases Institute, Korea University Guro Hospital, #148, Gurodong-ro, Guro-gu, Seoul 08308, Korea; chlthakd1029@naver.com (S.C.), dakjul@korea.ac.kr (H.-J.K.); 2 Department of Neurosurgery, National Health Insurance Service Ilsan Hospital, #100, Ilsan-ro, Ilsan-donggu, Goyang-si, Gyeonggi-do 10444, Korea; ulove07@nhimc.or.kr; 3 Department of Orthopedic Surgery, College of Medicine, Korea University, Anam-dong, Seongbuk-gu, Seoul 02841, Korea; lco20@hanmail.net; 4 Department of Systems Biotechnology, Chung-Ang University, Anseong-si, Gyeonggi-do 17546, Korea; luchiatkfkd@naver.com (H.-S.J.); minjiseon310@gmail.com (J.S.M.)

**Keywords:** icariin, nanodiamonds, MC3T3-E1 cells, osteogenesis promotion

## Abstract

Nanodiamonds (NDs) have been used as drug delivery vehicles due to their low toxicity and biocompatibility. Recently, it has been reported that NDs have also osteogenic differentiation capacity. However, their capacity using NDs alone is not enough. To significantly improve their osteogenic activity, we developed icariin (ICA)-functionalized NDs (ICA-NDs) and evaluated whether ICA-NDs enhance their in vitro osteogenic capacity. Unmodified NDs and ICA-NDs showed nanosized particles that were spherical in shape. The ICA-NDs achieved a prolonged ICA release for up to 4 weeks. The osteogenic capacities of NDs, ICA (10 μg)-NDs, and ICA (50 μg)-NDs were demonstrated by alkaline phosphatase (ALP) activity; calcium content; and mRNA gene levels of osteogenic-related markers, including ALP, runt-related transcript factor 2 (RUNX2), collagen type I alpha 1 (COL1A1), and osteopontin (OPN). In vitro cell studies revealed that ICA (50 μg)-ND-treated MC3T3-E1 cells greatly increased osteogenic markers, including ALP, calcium content, and mRNA gene levels of osteogenic-related markers, including ALP, RUNX2, COL1A1, and OPN compared to ICA (10 μg)-NDs or ND-treated cells. These our data suggest that ICA-NDs can promote osteogenic capacity.

## 1. Introduction

Bone tissues are composed of organic substances (i.e., collagen fibers) and inorganic components (i.e., hydroxyapatite ingredients) and they possess a high self-regenerative capacity as part of their healing response to fracture or injury. Nonetheless, large defects caused by serious damage or disease (e.g., trauma, infection, tumor resection, or arthritis) can cause bones to lose their self-renewal activity and eventually require surgical intervention [1,2]. As a gold standard approach, autologous bone grafts collect bone from a patient and implant it in the site of the defect [3]. Allografts have the advantages of easy use, an excellent safety profile, availability in different sizes and shapes, and the absence of the donor-site morbidity [4]. In addition, frozen bone and freeze-dried bone allografts have advantages, such as preservation of osteoinductive proteins, reduction of primary infection, and microbial contaminations [5,6,7]. Nevertheless, their use is severely hindered by limitations such as pain, limited supply, rejection, and disease transmission [8,9]. Alloplastic materials have been also applied due to their excellent productivity and scaffold function. They are produced by combining beta-tricalcium phosphate (β-TCP), hydroxyapatite (HAp), and/or calcium silicate [10,11,12]. However, the lack of their osteoinductivity leads to limited applications to only small or contained defects [13]. Xenogenic materials are mostly stable and biocompatible, and thus they have been used for treating various bone defects because they are similar or identical to the physicochemical properties of human bone [14]. However, several disadvantages, such as tissue reactions to xenografts and the complicated procedures regarding the removal of immunologically active cells and pathogens should be addressed [15].

Horny goat weed, also known as *Epimedii herba* or *Ying Yang Huo*, is extensively used to treat various diseases, including heart disease, osteoporosis, and rheumatism [16,17]. Icariin (ICA), one of the pharmacologically dynamic ingredients extracted from horny goat weed, possesses various therapeutic abilities, including neuroprotective, cardioprotective, anti-inflammatory, and anticancer actions [18,19,20]. Researchers have also shown an interest in the osteoinductive effects of ICA for bone tissue regeneration and the treatment of postmenopausal osteoporosis. ICA achieved the gene expression of osteogenic-related markers, including RUNX2 (runt-related transcription factor 2)/Id-1 (inhibitor of DNA-binding 1), in both pre-osteoblastic cells and osteoblasts [21]. ICA increased the proliferation of rat bone marrow derived mesenchymal stem cells (BMSCs) via extracellular signal-regulated kinase (ERK) and p38 mitogen-activated protein kinase (MAPK) pathways [22]. ICA can also induce early and late stage osteogenic markers (i.e., alkaline phosphatase (ALP), collagen type I (COL I), and the mineralization) of osteoblasts [23,24,25]. Feng et al. [24] reported that ICA not only significantly increased ALP, but also markedly decreased a bone resorption marker, carboxy-terminal collagen cross-links, compared with alendronate. Other studies showed that ICA inhibited the titanium (Ti) particles-stimulated bone resorption in vitro and decreased bone loss in a calvarial defect model induced by Ti particle–induced osteolytic sites in vivo [26,27]. Recently, our groups fabricated β-cyclodextrin-alginate (β-CD-ALG) conjugate to deliver ICA and confirmed the osteogenic activity. However, this nanocarrier has the limitation that the amount of ICA is able to deliver is relatively low [28].

To optimize the various advantages of ICA mentioned above, we used nanodiamonds (NDs) as a carrier in this study. Since the 1990s, nanomaterials have played an important role in a powerful therapeutic platform that combines therapeutic and diagnostic modalities. NDs are part of the carbon-based family that also includes fullerenes, carbon nanotubes, and graphene [29]. Recently, NDs have served as a versatile platform for biomedical applications owing to their low toxicity, adequate compatibility, and large surface area [29,30].

NDs as the delivery vehicle can easily be functionalized to connect with a variety of drugs or biomolecules via non-covalent and covalent bonds [31,32]. Moreover, NDs combined with various polymers could be used as regeneration scaffolds for bone defects. Zhang et al. [33] fabricated bone scaffolds containing PLLA and octadecylamine-coated NDs using a solution casting method. The proliferation of osteoblasts cultured on those scaffolds increased for up to 7 days, indicating that the scaffolds were not cytotoxic. Moore et al. [34] used electrostatic interactions to fabricate BMP-2-coated and bFGF-coated NDs to induce bone formation. NDs with both proteins induced the proliferation of osteoblasts and enhanced the differentiation of C2C12 cells. Ryu et al. [35] reported that alendronate-conjugated NDs showed enhanced ALP in MC3T3-E1 cells compared with unmodified NDs. More recently, lactoferrin delivery using NDs induced the osteogenic differentiation of cells and suppressed reactive oxygen species and inflammation [36].

The surfaces of scaffolds for treating bone defects have been modified with nanosized materials to improve bone tissue regeneration. Prior to the application of NDs on the surfaces of scaffolds, we tried to improve their osteogenic activity by functionalizing them with ICA. Thus, in this work, we developed ICA-functionalized NDs with osteogenic capacity and examined the in vitro osteogenic capacity of ICA-NDs by determining the ALP, the calcium levels, and the osteogenic-related mRNA levels in MC3T3-E1 cells.

## 2. Materials and Methods

### 2.1. Materials

ICA, carboxylated NDs (cNDs), and 2-morpholinoethanesulfonic acid (MES) were purchased from Tokyo Chemical Industry (TCI, Tokyo, Japan). Dopamine hydrochloride (termed as DOPA), Tris-hydrochloride (Tris-HCl), ethanol (EtOH), *p*-nitrophenyl phosphate, sodium hydroxide (NaOH), *o*-cresolphthalein complexone, 8-hydroxy-quindine, and 2-amino-2-methyl-1-propanol (AMP) were obtained from Sigma-Aldrich (St. Louis, MO, USA). Fluorescein isothiocyanate (FITC) and 4-6-diamidino-2-phenylindole (DAPI) were procured from Thermo Fisher Scientific (Waltham, MA, USA). Phosphate-buffered saline (PBS) and Dulbecco’s modified eagle’s medium (DMEM) were purchased from Welgen Inc (Gyeongsan, Korea). For in vitro drug release study, dialysis membrane (MWCO: 6–8 kDa) was obtained from Spectrum Laboratories Inc. (Rancho Dominguez, CA, USA). Cell counting kint-8 (CCK-8) reagent was purchased from Dojindo Inc. (Kumamoto, Japan).

### 2.2. Icariin-Functionalized Nanodiamonds

To functionalize NDs with ICA, the surfaces of NDs were modified as described in the previous study [17]. Ten milligrams of cNDs were dispersed in 10 mM Tris-HCl (pH 8.5) containing DOPA (5 mg/mL) and then mildly agitated under in the dark. After overnight reaction, DOPA-modified NDs (named NH_2_-NDs) were washed three times with deionized water (DW) and then lyophilized for 3 days. The obtained NH_2_-NDs were further functionalized by treating with ICA (10 or 50 μg/mL) dissolved in a co-solvent of EtOH:Tris-HCl (4:6, *v*/*v*) for 24 h. The obtained two kinds of ICA-functionalized NDs (termed ICA (10 μg)-NDs and ICA (50 μg)-NDs) was washed twice with DW and lyophilized for 3 days.

### 2.3. Characterization of Plain NDs and Modified NDs

For the scanning electron microscopy (SEM) analysis, NDs, NH_2_-NDs, ICA (10 μg)-NDs, and ICA (50 μg)-NDs were dispersed in EtOH using a bath-type sonicator (Powersonic 405, Hwashin Tech Co., Seoul, Korea) and then dropped on a glass coverslip and air-dried. After coating with platinum, their morphological shapes were observed with field-emission SEM (S-4700, Hitachi, Japan). For their particle size analysis, 0.1 mg/mL of each sample was suspended in DW by sonicating at 4 °C for 1 h with a bath type sonicator. Then, their particle sizes and zeta potential values were determined with a dynamic light scattering (DLS) instrument (Malvern Matersizer 3000, Malvern Panalytical Ltd., Malvern, UK) equipped with a 633 nm He–Ne laser. The surface modifications of all samples were investigated using an attenuated total reflectance-Fourier transform infrared (ATR-FTIR) spectroscope (Thermo Electron Corp., Madison, WI, USA). All the ATR-FTIR spectra were scanned in the range between 4000 and 400 cm^−1^ with a 4 cm^−1^ resolution. The surface chemical compositions of NDs, NH_2_-NDs, and two kinds of ICA-NDs were analyzed using the X-ray photoelectron spectrometer ESCALAB 250 ultrahigh vacuum (1 × 10^–9^ bar) instrument (Thermo Scientific Inc., Waltham, UK) equipped with an Al Kα X-ray source. Al Kα X-ray source (1486.6 eV photons, beam size: 500 μm) was used for acquiring C1s, N1s, and O1s signals of all samples.

### 2.4. ICA Release

Ten micrograms of ICA (10 μg)-NDs and the same of ICA (50 μg)-NDs, respectively, were dispersed in PBS (1 mL, pH 7.4) using a bath-type sonicator. After the dispersed samples were carefully transferred into a dialysis membrane bag, the membrane bag was soaked into 15 mL of conical tube containing 5 mL of PBS and then oscillated at 100 rpm and 37 °C. At different incubation time points, such as 1, 5, 9, 12 h, 1, 3, 5, 7, 14, 21, and 28 days, the PBS solution was collected and the same volume of fresh PBS solution was changed. The amount of ICA released was determined at 290 nm using a multi-mode plate reader (Thermo Scientific, MA, USA). Each group was *n* = 4. This experiment was replicated thrice.

### 2.5. Cell Viability Analysis

To examine the cytotoxicity of NDs and two kinds of ICA-NDs, MC3T3-E1 cells (murine pre-osteoblast cell line, Korean Cell Line Bank, Seoul, Korea) were seeded into a 96-well culture plate at a density of 1 × 10^4^ cells per well. The cells were further cultured in DMEM overnight. Then, the cells were treated with NDs and two ICA-NDs at 100 μg/mL concentration. After exposure for 24 h or 48 h, the cells were carefully washed with PBS. After adding CCK-8 reagent into each well, the cells were further incubated for 1 h at 37 °C. Afterwards, the optical density at 450 nm was measured using a multi-mode plate reader. Cell viability was represented as the percentage of viable cells compared to the control group. All experiments per time point were repeated three times.

### 2.6. Infiltration of Nanodiamonds into Cells

To investigate whether NDs can be internalized into MC3T3-E1 cells, FITC-labeled NDs were prepared via chemical reaction of NH_2_-NDs (10 mg) with FITC in MES buffer at room temperature in the dark for 24 h. Then, FITC-labeled NDs were collected by centrifugation at 8000 rpm for 15 min and freeze-dried for 3 days. For observation of the cellular uptake of FITC-labeled NDs into cells, the cells (1 × 10^4^ cells/well) were seeded and incubated on 12-mm diameter microscope cover glass (Paul Marienfeld GebH and Co. KG, Lauda-Königshofen, Germany) overnight. Afterwards, the cells were treated with FITC-labeled NDs at a concentration of 100 μg/mL. After 4 h treatment, the cells were fixed with 3.7% paraformaldehyde for 30 min. Additionally, after the cell nuclei were counter-stained with DAPI for 30 min, the cells were imaged using a confocal laser scanning microscope (CLSM; LSM700, Zeiss, Hsu Koehn, Germany).

### 2.7. Early Osteogenic Differentiation Marker Analysis

For determining ALP as an early osteogenic differentiation marker, MC3T3-E1 cells (1 × 10^5^ cells/well) were incubated on 24-well plates and treated with NDs, ICA (10 μg)-NDs, and ICA (50 μg)-NDs) at a concentration of 100 μg/mL. After treatment for 3 or 9 days, the cells were carefully washed with PBS and they were lysed using a lysis buffer (1× RIPA buffer) which was composed of Tris-HCl (50 mM, pH 7.4), NaCl (150 mM), deoxycholic acid (0.25%), tergitol-type-40 (1%, NP-4), ethylenediaminetetraacetic acid (EDTA, 1 mM), and protease/phosphatase inhibitors (phenylmethylsulfonyl fluoride (1 mM), sodium orthovanadate (1 mM), sodium fluoride (1 mM), aprotinin (1 μg/mL), leupeptin (1 μg/mL), and pepstatin (1 μg/mL)). After the lysed cells were centrifuged at 13,500 rpm for 10 min at 4 °C, the supernatant collected from each group was reacted with *p*-nitrophenyl phosphate at 37 °C for 30 min, and 1 N NaOH solution (500 μL) was added to terminate the reaction. During such reaction, *p*-nitrophenyl phosphate was converted into *p*-nitrophenol. ALP activity was determined by measuring the absorbance at 405 nm with a multi-mode plate reader. All experiments per time point were repeated three times.

### 2.8. Late Osteogenic Differentiation Marker Analysis

To assess the expressions of late osteogenic differentiation markers, we conducted calcium deposition analysis. The cells and sample treatments were the same as described in the ALP activity study. After exposure of each sample for 7 or 21 days, the cells were carefully washed twice with PBS. Then, the cells were treated with 0.5 N HCl and further incubated at 37 °C under shaking at 100 rpm overnight. Afterwards, each group was treated with a calcium standard solution, followed by the addition of a mingling solution containing *o*-cresolphthalein, complexone, and 8-hydroxy-quindine for 1 min and the further addition of 2-amino-2-methyl-1-propanol buffer. The resulting solutions were mixed well for 15 min and carefully transferred into 96-well plates. Finally, the absorbance was measured at 575 nm with a multi-mode plate reader. All experiments per time point were repeated three times.

### 2.9. Evaluation of Osteogenic-Related Genes

To further compare the osteogenic activities of NDs and two types of ICA-NDs, the mRNA levels of osteogenic-related markers, including ALP, RUNX2, collagen type I (COL1A1), and osteopontin (OPN), were evaluated in the cells with the real-time PCR. Cells (1 × 10^5^ cells) were plated onto each well of 24-well culture plates and exposed to each sample (100 μg/mL). On days 7 and 21, after harvesting the cells, the total RNA was obtained using a RNeasy Plus mini kit. After establishing the RNA concentrations, cDNA was reverse-transcribed from the total RNA (1 μg) using AccuPower RT PreMix. The primer sequences of the target genes are described in the Supplementary Materials (Table S1). PCR amplification and real-time PCR were performed using an ABI7300 Real-Time PCR System (Applied Biosystems, USA). All experiments per time point were repeated three times.

### 2.10. Statistical Analysis

The data represent means ± standard deviations; mean values were compared among many groups by one-way ANOVA (Systat software, Chicago, IL, USA). Comparisons of ICA (10 μg)-NDs versus NDs, ICA (50 μg)-NDs versus NDs, and ICA (10 μg)-NDs versus ICA (50 μg)-NDs were performed. *p* values less than 0.01 and 0.05 were considered statistically significant.

## 3. Results

### 3.1. Characterizations

**Figure 1:**
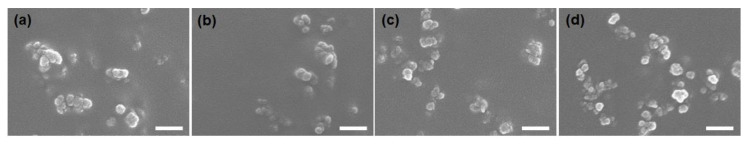
SEM images of (**a**) NDs, (**b**) NH_2_-NDs, (**c**) ICA (10 μg)-NDs, and (**d**) ICA (50 μg)-NDs. Scale bar: 200 nm.

To develop the ICA-NDs, the surfaces of cNDs were modified with dopamine and then coated with ICA. The ICA loading efficiency was 6.10 ± 0.90 μg (61.00% ± 8.97%) for the ICA (10 μg)-NDs and 33.43 ± 2.91 μg (66.85% ± 5.82%) for the ICA (50 μg)-NDs. SEM images show the morphologies of the cNDs, NH_2_-NDs, ICA (10 μg)-NDs, and ICA (50 μg)-NDs (Figure 1). Each ND sample was spherical and the individual particle sizes of each group in dried state ranged from approximately 30 to 50 nm. The mean hydrodynamic size distributions and polydispersity indexes of the cNDs, NH_2_-NDs, ICA (10 μg)-NDs, and ICA (50 μg)-NDs (detected by dynamic light scattering (DLS)) were 156.40 ± 75.50 nm and 0.183, 157.30 ± 135.30 nm and 0.223, 161.20 ± 110.40 nm and 0.205, and 161.20 ± 110.40 nm and 0.205, respectively. Compared to sizes in SEM data, the mean hydrodynamic sizes of each group were much bigger due to their aggregation in aqueous solution. The zeta potential values were −32.03 ± 1.24 mV for cNDs, −30.53 ± 1.70 mV for NH_2_-NDs, −32.57 ± 0.31 mV for ICA (10 μg)-NDs, and −35.90 ± 0.17 mV for ICA (50 μg)-NDs. After modification of cNDs with DOPA, zeta potential value increased, indicating the DOPA was successfully modified on the surfaces of cNDs. However, the reactions of NH_2_-NDs with ICA led to the decrement of zeta potential values due to the successful functionalization of ICA on NH_2_-NDs.

**Figure 2:**
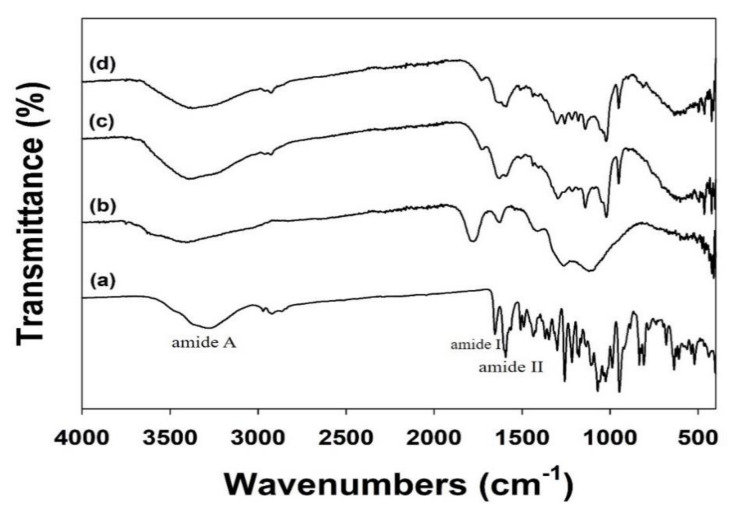
FTIR spectra of (**a**) ICA, (**b**) NDs, (**c**) ICA (10 μg)-NDs, and (**d**) ICA (50 μg)-NDs.

We conducted an ATR-FTIR investigation to assess the ICA coating on the ND surfaces (Figure 2). In the ATR-FTIR, ICA showed major characteristic peaks at 1652 cm^−1^ (amide I, C=O stretching), 1598 cm^−1^ (amide II, C-N stretching), and 1073 cm^−1^ (C-O-C stretching). After coating the NDs with ICA, these distinctive ATR-FTIR spectral peaks of ICA were also detected in the ATR-FTIR of the ICA (10 μg)-NDs and ICA (50 μg)-NDs.

To confirm the functionalization of ICA on the NDs, we conducted an XPS analysis (Table 1). An outstanding increase in the N1s component was shown for the NH_2_-NDs anchored by DOPA. Compared with the NH_2_-NDs, the two types of ICA-NDs (10 and 50 μg) exhibited a small decline in the N1s component and an increase in the C1s component, indicating the successful dopamine anchorage on the NDs and ICA functionalization on the NH_2_-NDs.

### 3.2. In Vitro ICA Release

**Figure 3:**
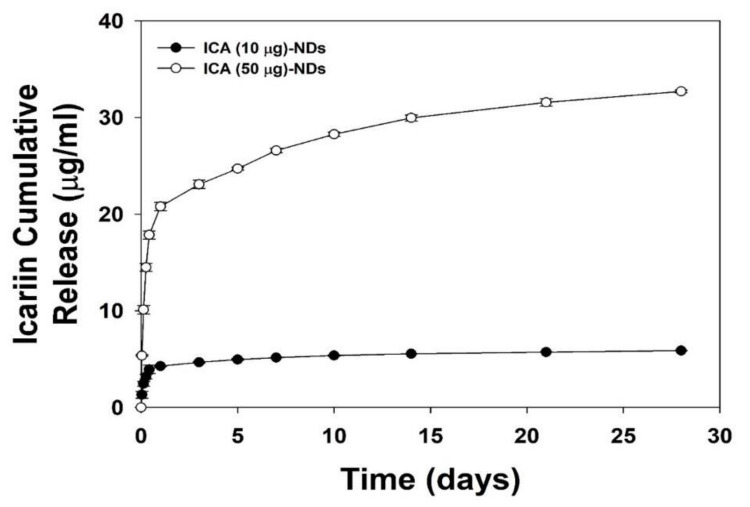
In vitro cumulative ICA release from the ICA (10 μg)-NDs and ICA (50 μg)-NDs (*n* = 4/group).

In vitro ICA releases from the ICA (10 μg)-NDs and ICA (50 μg)-NDs for 28 days are shown in Figure 3. On day 1, the ICA (10 μg)-NDs released 4.27 ± 0.03 μg (72.09% ± 0.56%), and the ICA (50 μg)-NDs released 20.79 ± 0.43 μg (62.20% ± 1.30%). Over the course of 28 days, the ICA (10 μg)-NDs and ICA (50 μg)-NDs released 5.87 ± 0.02 μg (98.30% ± 0.34%) and 32.70 ± 0.14 μg (97.82% ± 0.42%) of ICA, respectively.

### 3.3. Cytotoxic Test and Cellular Internalization

**Figure 4:**
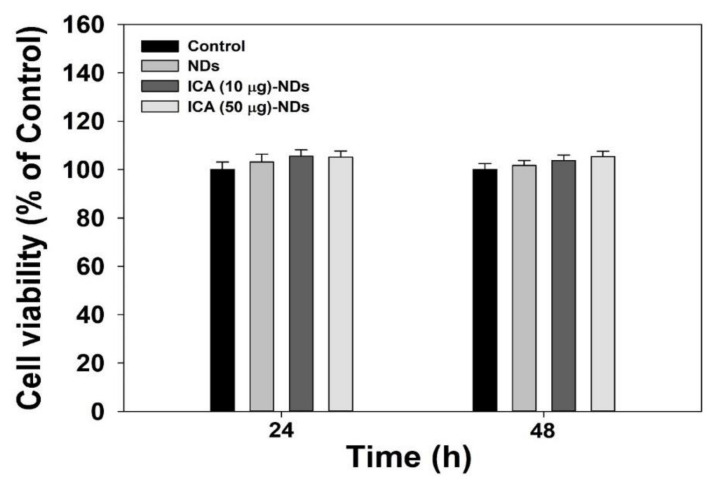
Cytotoxic effects of NDs, ICA (10 μg)-NDs, and ICA (50 μg)-NDs on MC3T3-E1 cells (*n* = 4/group).

Before estimating the osteogenic differentiation activity using NDs with and without ICA, a cytotoxicity investigation is essential. The cytotoxic effects of NDs with and without ICA on MC3T3-E1 cells were studied for 48 h (Figure 4). Cell viability after treatment with each sample remained more than 95% as prevalent as in the control group for 48 h. Thus, no sample produced any obvious cytotoxicity in the MC3T3-E1 cells.

**Figure 5:**
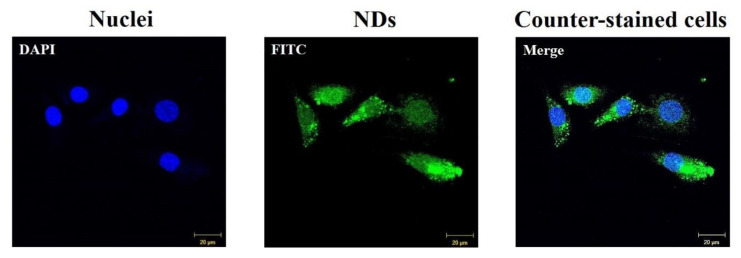
CLSM images of intracellular uptake of FITC-labeled NDs after 4 h of cultivation. Scale bar: 20 μm.

The results from the test of the intracellular uptake of FITC-labeled NDs are denoted in Figure 5. After 4 h of cultivation, the FITC-labeled NDs were clearly delivered into the cytoplasms of the MC3T3-E1 cells.

### 3.4. Early Osteogenic Differentiation Marker Analysis

**Figure 6:**
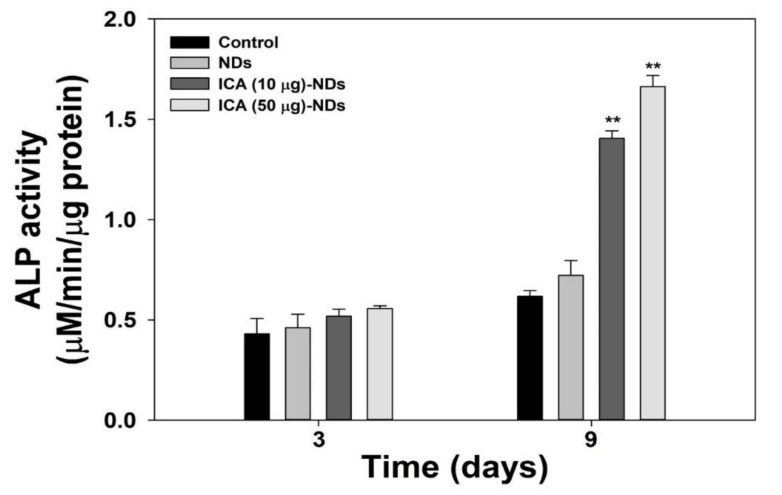
ALP levels of MC3T3-E1 cells cultivated with NDs, ICA (10 μg)-NDs, and ICA (50 μg)-NDs after 3 and 9 days of culture (*n* = 4/group). ** *p* < 0.01.

To verify the early osteogenic differentiation markers, we tested the ALP activity. The ALP activity increased with the cultivation time for all the experimental groups (Figure 6). After 3 days, no big differences in the ALP levels of the MC3T3-E1 cells were observed among any groups. After 9 days, however, the ALP activity in the cells treated with ICA-NDs was remarkably greater than in the cells that received NDs (** *p* < 0.01).

### 3.5. Late Osteogenic Differentiation Marker Analysis

**Figure 7:**
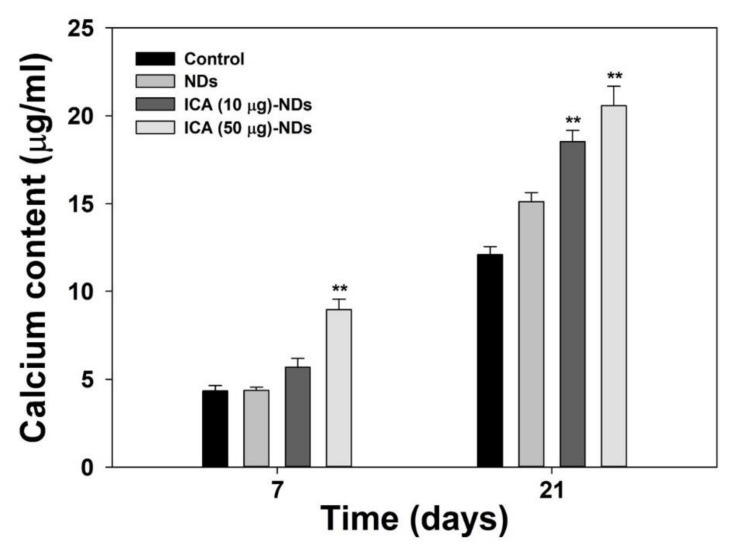
The calcium levels in MC3T3-E1 cells treated with NDs, ICA (10 μg)-NDs, and ICA (50 μg)-NDs after 7 and 21 days of culture (*n* = 4/group). ** *p* < 0.01.

For the late osteogenic differentiation analysis, we conducted a calcium deposition test. Figure 7 reveals the amounts of calcium in MC3T3-E1 cells treated with NDs with and without ICA on days 7 and 21 of incubation. The amounts of calcium in MC3T3-E1 cells treated with ICA-NDs increased for up to 21 days. On day 7, the calcium content in the cells treated with ICA (50 μg)-NDs was outstandingly different from the amount deposited by cells treated with bare NDs (** *p* < 0.01). On day 21, calcium content in MC3T3-E1 cells treated with ICA-NDs was notably greater than that in MC3T3-E1 cells cultured with NDs (** *p* < 0.01).

### 3.6. Osteogenic-Related Genes

**Figure 8:**
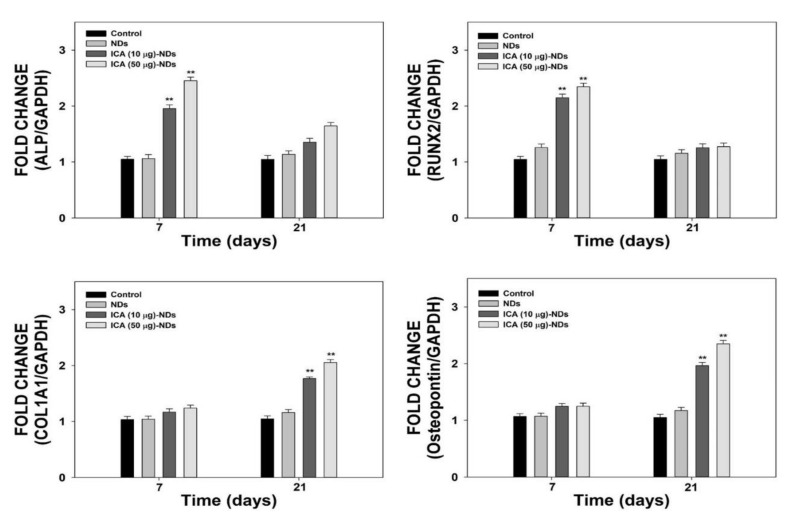
The mRNA levels of ALP, RUNX2, COL1A1, and OPN in cells treated with NDs, ICA (10 μg)-NDs, and ICA (50 μg)-NDs after 7 and 21 days of incubation (*n* = 4/group). ** *p* < 0.01.

To assess the osteogenic differentiation influences of NDs with and without ICA, we estimated the mRNA levels of osteogenic-related markers such as ALP, RUNX2, COL1A1, and OPN, using real-time PCR after 7 and 21 days of cultivation (Figure 8). The COL1A1 and OPN gene levels in the cells incubated with ICA-NDs increased substantially in a time- and concentration-dependent manner. On day 7, the levels of the four genes in the cells treated with ICA-NDs were markedly higher than those in cells treated with NDs (** *p* < 0.01). However, the ALP and RUNX2 gene levels in all the cells were downregulated on day 21, suggesting a shift in the cellular procedure to mineralization. In contrast, the expression levels of COL1A1 and OPN in the cells treated with ICA-NDs were much higher than in cells that received NDs (** *p* < 0.01).

## 4. Discussion

The objective in this study was to demonstrate whether ICA-NDs could boost the osteogenic capacity of MC3T3-E1 cells. To fabricate the ICA-NDs, ICA was applied to functionalize ND surfaces via DOPA anchoring. NDs with and without ICA were investigated by TEM, and the DLS covered between 150 and 180 nm. Kim et al. [36] investigated the release of osteogenic potential protein, viz., LF, from NDs via ionic interaction between the amine group of the LF and the carboxylic groups of the NDs. They observed that LF release was sustained for up to 7 days. Consistent with that previous study, we discovered that the ICA-NDs showed sustained and controlled release behavior for 28 days. Thus, NDs as drug delivery carriers should be capable of both short-term and long-term sustained drug release.

Zhang et al. [37] fabricated an ICA-containing porous β-TCP ceramic (ICA/β-PTCP) scaffold. The ICA/β-PTCP not only supported the proliferation/differentiation of Ros 17/28 cells (rat osteoblast-like cells) but also promoted more new bone formation after implantation into the backs of rats than β-PTCP did. Many researchers have discovered that ICA combined with various bone substitutes, such as a PHBV scaffold, PLGA/β-TCP scaffold, and calcium phosphate cement, markedly enhanced cell growth in vitro and promoted osteogenesis and angiogenesis in vivo [38,39,40].

Due to the excellent qualities of ICA just described, we demonstrated the osteogenic capacities of ICA-NDs in MC3T3-E1 cells by analyzing an early differentiation marker, a late differentiation marker, and osteogenic-related gene markers. The osteogenic differentiation of MSCs is classified into three phases [41]. In vitro, osteogenic differentiation happens for one month and creates differentiated osteoblasts. ALP is an early marker of osteoblast differentiation [42]. The mRNA and protein expressions of ALP appear between days 5 and 14 of osteoblast differentiation [43]. After the ALP has reached its peak, the level begins to decrease. The ALP levels of MC3T3-E1 cells treated with NDs in the presence and absence ICA were investigated after 3 and 9 days of culture. The ALP levels of the cells treated with NDs, ICA (10 μg)-NDs, and ICA (50 μg)-NDs improved time- and dose-dependently. No significant difference was found between the ALP levels of MC3T3-E1 cells cultured with NDs and ICA-NDs after 3 days. However, the ALP level of MC3T3-E1 cells treated with ICA-NDs was remarkably higher than for those treated with NDs after 9 days. Calcium deposition levels, a key component of osteogenesis, have been used to confirm the late osteogenic differentiation of osteoblasts [44]. The calcium deposition levels of MC3T3-E1 cells cultured with NDs with and without ICA were assessed after 7 and 21 days of culture. Calcium sedimentation by MC3T3-E1 cells treated with ICA-NDs at two ICA concentrations increased compared with those in cells treated with NDs after 7 and 21 days. Wu et al. showed that the ALP level of BMSCs cultured on new ICA-loaded hybrid hydroxyapatite granules with micro/nano pores was significantly enhanced [40]. Gong et al. reported improved ALP and calcium levels in MC3T3-E1 cells cultured on an ICA-loaded polycaprolactone (PCL)/gelatin fibrous matrix via the release of ICA [45]. Those previous results are consistent with our results and suggest that ICA elevates ALP and calcium levels.

To further examine osteogenic differentiation, we evaluated the mRNA levels of four osteogenic-related genes (ALP, RUNX2, COL1A1, and OPN) in the cells treated with NDs in the presence and absence of ICA using real-time PCR after 7 and 21 days of incubation. RUNX2, known as a master gene for osteoblast differentiation, regulates a downstream gene that determines the phenotype of osteoblasts, and its gene is expressed at the early stages during osteogenic differentiation along with the ALP gene [46,47]. The organic part of the bone extracellular matrix (ECM) is composed of collagen type I. It also contains non-collagenous bone proteins, including OPN and osteocalcin, which are critical for coordinating organic matrix and bone minerals [43,47,48]. OPN, a glycosylated phosphoprotein localized in bone and dentin, is secreted by osteoblasts into a mineralized ECM during bone development [47]. The expression levels of four osteogenic-related marker genes in the cells treated with NDs in the presence and absence of ICA did not increase in a time-dependent manner. ICA-NDs significantly improved ALP and RUNX2 gene expression compared with bare NDs at 7 days, but their mRNA expression was downregulated on day 21 due to reaching the mineralization stage [43,46,47]. The COL1A1 and OPN expression levels in MC3T3-E1 cells treated with ICA-NDs were remarkably higher than those in MC3T3-E1 cells treated with NDs on day 21. The expression of RUNX2/Id-1 was reported to be achieved by ICA in both pre-osteoblastic MC3T3-E1 cells and osteoblasts. Previous studies reported that ICA treatment not only enhanced COL I expression in both rBMSCs and MC3T3-E1 cells, but also increased the OPN level in MC3T3-E1 cells [16,23,45]. Recently, it was reported that miR-153 promoted the mRNA and protein levels of RUNX2 during the osteoblast differentiation of MC3T3-El cells [49]. Interestingly, RUNX2 levels were increased by treating ICA in miR-153 inhibitor-transfected MC3T3-E1 cells, indicating that ICA may regulate the osteoblast differentiation. These results imply that ICA has effects in both early and late osteoblast differentiation. For clinical bone regeneration treatment, ICA alone must overcome several limitations, including the induction of cytotoxicity and apoptosis in several cell types and its poor biocompatibility [50]. To overcome those problems, we used ICA-NDs to improve the osteogenic capacity of MC3T3-E1 cells by releasing ICA from the NDs. Although the fabricated ICA-NDs should be tested more using in vivo animal models, the combinations of alloplastic materials with ICA-NDs will improve bone generative effects on defect models.

## 5. Conclusions

The present study reported that the osteogenic capacity of MC3T3-E1 cells could be promoted via ICA-NDs. The ICA-NDs showed sustained release of ICA and improved the osteogenic capacity of MC3T3-E1 cells by inducing ALP action and enhancing calcium sedimentation. Furthermore, they clearly enhanced ALP and RUNX2 expression (early osteogenic-related genes) and COL1A1 and OPN levels (late osteogenic-related genes) through the sustained release of ICA from the NDs. Therefore, we believe that ICA-NDs will have great potential to improve bone defects and diseases.

## Figures and Tables

**Table 1:** Elemental compositions of unmodified NDs and modified NDs by XPS analysis.

	Elements	C1s (%)	N1s (%)	O1s (%)	Total (%)
Groups					
NDs		91.60	-	8.40	100
NH_2_-NDs		89.00	2.15	8.85	100
ICA (10 μg)-NDs		89.20	1.90	8.90	100
ICA (50 μg)-NDs		89.55	1.73	8.72	100

## Supplementary Materials

The following are available online at https://www.mdpi.com/2079-4991/10/10/2071/s1. Table S1: Real-time PCR primer sequences of the osteogenic-related genes.
