# Supplementary material for: Icariin-Functionalized Nanodiamonds to Enhance Osteogenic Capacity In Vitro
Source: Nanomaterials (Basel). 2020 Oct 20;10(10):2071. doi: 10.3390/nano10102071 (PMC7589593; doi:10.3390/nano10102071)
Supplement: Supplementary file 1 [file nanomaterials-10-02071-s001.pdf]

nanomaterials

Supplementary Materials

Icariin-Functionalized Nanodiamonds to Enhance Osteogenic Capacity In Vitro

Somang Choi [1,†], Sung Hyun Noh [2,†], Chae Ouk Lim [3,†], Hak-Jun Kim [1], Han-Saem Jo [4], Ji Seon Min [4], Kyeongsoon Park [4,*] and Sung Eun Kim [1,*]

[1] Department of Orthopedic Surgery and Rare Diseases Institute, Korea University Guro Hospital, #148, Gurodong-ro, Guro-gu, Seoul 08308, Republic of Korea; chlthakd1029@naver.com (S.C.), dakjul@korea.ac.kr (H.-J.K.)
[2] Department of Neurosurgery, National Health Insurance Service Ilsan Hospital, #100, Ilsan-ro, Ilsan-donggu, Goyang-si, Gyeonggi-do 10444, Republic of Korea; ulove07@nhimc.or.kr
[3] Department of Orthopedic Surgery, College of Medicine, Korea University, Anam-dong, Seongbuk-gu, Seoul 02841, Republic of Korea; lco20@hanmail.net
[4] Department of Systems Biotechnology, Chung-Ang University, Anseong-si, Gyeonggi-do 17546, Republic of Korea; luchiatkfkd@naver.com (H.-S.J.); minjiseon310@gmail.com (J.S.M.)
† S.C., S.H.N. and C.O.L. contributed equally to this work
* Correspondence: kspark1223@cau.ac.kr (K.P.); sekim10@korea.ac.kr (S.E.K.); Tel.: +82-31-670-3357 (K.P.); +82-2-2626-1999 (S.E.K.)

Table S1. Real-time PCR primer sequences of the osteogenic-related genes.

Gene	Primer (Forward)	Primer (Reverse)
ALP	GTG GAA GGA GGC AGA ATT GAC CA	AGG CCC ATT GCC ATA CAG GAT GG
RUNX2	ATG GCA TCA AAC AGC CTC TTC AGC A	CGT GGG TTC TGA GGC GGG ACA CC
COL1A1	CCC TGG AAA GAA TGG AGA TG	CCA CTG AAA CCT CTG TGT CC
OPN	GAG GGC TTG GTT GTC AGC	CAA TTC TCA TGG TAG TGA GTT TTC C

© 2020 by the authors. Licensee MDPI, Basel, Switzerland. This article is an open access article distributed under the terms and conditions of the Creative Commons Attribution (CC BY) license (http://creativecommons.org/licenses/by/4.0/).
